# Cadherin-Mediated Cell Adhesion Is Critical for the Closing of the Mouse Optic Fissure

**DOI:** 10.1371/journal.pone.0051705

**Published:** 2012-12-11

**Authors:** Shuyi Chen, Brandy Lewis, Andrea Moran, Ting Xie

**Affiliations:** 1 Stowers Institute for Medical Research, Kansas City, Missouri, United States of America; 2 Department of Cell Biology and Anatomy, University of Kansas Medical Center, Kansas City, Kansas, United States of America; University of Birmingham, United Kingdom

## Abstract

Coloboma is a congenital disease that contributes significantly to childhood blindness. It results from the failure in closing the optic fissure, a transient opening on the ventral side of the developing eye. Although human and mouse genetic studies have identified a number of genes associated with coloboma, the detailed cellular mechanisms underlying the optic fissure closure and coloboma formation remain largely undefined. N-cadherin-mediated cell adhesion has been shown to be important for the optic fissure closure in zebrafish, but it remains to be determined experimentally how cell-cell adhesions are involved in the mammalian optic fissure closing process. α-catenin is required for cell adhesion mediated by all of the classic cadherin molecules, including N-cadherin. In this study, we used the Cre-mediated conditional knockout technique to specifically delete *α-catenin* from the developing mouse eye to show that it is required for the successful closing of the optic fissure. In *α-catenin* conditional mutant optic cups, the major cell fates, including the optic fissure margin, neural retina and retinal pigmented epithelium, are specified normally, and the retinal progenitor cells proliferate normally. However, adherens junctions components, including N-cadherin, β-catenin and filamentous actin, fail to accumulate on the apical side of *α-catenin* mutant retinal progenitor cells, where adherens junctions are normally abundant, and the organization of the neural retina and the optic fissure margin is disrupted. Finally, the *α-catenin* mutant retina gradually degenerates in the adult mouse eye. Therefore, our results show that α-catenin-mediated cell adhesion and cell organization are important for the fissure closure in mice, and further suggest that genes that regulate cell adhesion may underlie certain coloboma cases in humans.

## Introduction

Epithelial fusion is an important morphogenetic process frequently used to close originally open structures during embryogenesis and wound healing. It is also one of the key steps in vertebrate eye development. The development of the vertebrate eye begins with the bilateral evagination of the diencephalon in the early neurula, leading to the formation of the hollow tube-shaped structure, called the optic vesicle (OV). When it approaches the surface ectoderm, the OV begins to invaginate to form the optic cup (OC) [1]–[3]. The OV invagination has two important developmental consequences: Juxtaposition of the neural retinal (NR) layer atop the retinal pigmented epithelial (RPE) layer, and the formation of the optic fissure (OF). The NR progenitor cells on the inner layer rapidly proliferate and give rise to six types of neurons and Müller glial cells that are organized into three distinct cellular layers, while RPE progenitor cells on the outer layer provide supporting and protective roles for NR progenitors [1], [4]–[6]. Because of the asymmetric invagination orientation of the OV, a fissure on the ventral side of the developing retina forms, running from the distal end of the OC to the proximal junction with the forebrain [1], [7]–[9]. The OF provides the opening for the entry of the surrounding mesenchymal cells into the OC, which form hyaloid vessels for supplying blood to the developing retina. After the mesenchymal cells finish migration, the laterally growing edges of the OC at the OF margin align against each other to fuse and form a continuous OC, a process known as the OF closure [1], [7], [10]. The OF closure leaves a small opening at the center of the OC to form the optic disc, which provides an exit and guidance cues for the projecting axons of retinal ganglion cells toward the optic stalk to form the optic nerve connecting the eye to the brain.

When the OF closure fails, a permanent opening remains on the ventral side of the eye, the congenital abnormality known as coloboma. Coloboma contributes significantly to childhood blindness, occurring in isolation or within syndromes [7], [11]. Clinical epidemiology and genetic studies show that coloboma exhibits considerable genetic heterogeneity, variable expressivity and different degrees of penetrance, indicating that the OF closure is controlled by a complex molecular network [7], [11]. Human and mouse genetic studies have linked a number of genes with coloboma, many of which play important roles in retinal fate specification and patterning. For example, mutations in the genes that are important for the specification or maintenance of the ventral retina, including *Pax2*, *Vax1* and *Vax2*, produce the coloboma phenotype [12]–[15]. In addition, mutations in *Shh*, which promotes the proximal optic stalk fate but suppresses the distal retinal fate, have been found in coloboma patients, suggesting that the proper specification of the proximal-ventral retinal fate is critical for the proper OF closure [16]. In addition, RPE progenitors and peri-ocular mesenchymal cells also appear to play important roles in the regulation of the OF closure. RPE specific deletion of *β-catenin* disrupts proper RPE differentiation, resulting in the coloboma phenotype [17], while mutations in *Chd7* and *Pitx2*, which are expressed in mesenchymal cells and essential for their development, cause coloboma formation [18]–[21]. Finally, cell proliferation appears to be critical for the OF closure. A mutation in *Phactr4*, which encodes a negative regulator of cell proliferation, leads to the coloboma phenotype [22]. Although many genes have been linked to coloboma, their potential connections in the regulation of the OF closure are still largely unknown.

The OF closure is accompanied by cell morphological changes and rearrangement at the OF region. Cell-cell adhesion has been shown to be essential for cell morphological changes and rearrangement by providing structural connections between cells [23], [24]. In zebrafish, a mutation in *pac*, which encodes N-cadherin, causes tissue organization defects and the coloboma phenotype, providing direct evidence that adherens junctions (AJs) are important for eye patterning and morphogenesis [25]. However, it remains unclear if AJ requirement in the OF closure is conserved in mammals. In this study, we use the conditional knockout technique to specifically delete *α-catenin* from the developing mouse retina to examine its role in retinal development. α-catenin is a filamentous actin (F-actin) binding and bundling protein, and is one of the key components of the AJ complex [26]. Based on its ability to directly bind β-catenin and actin, α-catenin was originally thought to provide a static physical connection between AJs and the actin network. However, α-catenin has been recently shown to shuttle between cadherin/β-catenin complexes and F-actin, thus having regulatory roles in cell-cell adhesion and cytoskeleton organization [27], [28]. In this study, we show that α-catenin mediated cell-cell adhesion is essential for the OF closure.

## Materials and Methods

All animal work was performed in compliance with the protocols approved by the Institutional Animal Care and Use Committee at the Stowers Institute for Medical Research (SIMR). The following mice are used in this study: *Six3-Cre* [29] and *Ctnna1^fx^* [30]. Noon on the day at which a vaginal plug is found is referred to as embryonic day 0.5 (E0.5).

### Tissue preparation and immunohistochemistry

Embryos were fixed overnight in 4% formaldehyde, cryo-preserved with 15% sucrose followed by 30% sucrose, and frozen using a freezing bath (Thermo Scientific) with isopentane. Because the OF is a ventral-specific structure of the OC, most specimens were sectioned para-sagittally. The nasal-temporal orientation of the OC was determined based on the brain structures around the eye according to The Atlas of Mouse Development [31].

For immunohistochemistry, tissue sections were heated in citrate buffer (pH 6.0) at 95°C for 10 minutes. They were then incubated with the primary antibodies at 4°C overnight, and then with Alexa 488- or Alexa 568-conjugated goat or donkey secondary antibodies (Invitrogen) for 2 hours at room temperature. Finally, tissue sections were counter-stained with DAPI for 5 minutes, washed and mounted. The following antibodies were used: Mouse anti-α-catenin (Invitrogen), rabbit anti-β-Catenin (Invitrogen), rabbit anti-Pax2 (Invitrogen), mouse anti-Pax6 (Developmental Studies Hybridoma Bank), sheep anti-Vsx2 (Chemicon), mouse anti-Mitf (lab Vision), goat anti-pMLC (Santa Cruz), rabbit anti-N-cadherin (Santa Cruz), and mouse anti-Laminin (Sigma). Images were taken under either Leica SP2 or SP5 confocal microscope.

### BrdU incorporation assay

The time-mated mice were injected intraperitoneally with BrdU at 0.1 mg/g body weight two hours before sacrifice. Embryos were fixed, sectioned and immuostained for BrdU (Amersham) as described above. For each para-sagittal section of the OC, about 60–100 retina progenitor cells (based on DAPI staining) in the central region of nasal and temporal retinas and retinal cells within a 5-cell diameter from the margin of the OF were counted. For each genotype, four eyes from four mice were quantified. For statistical analysis, Student's t-test was applied.

## Results

### α-catenin Is Required for Controlling the OF Closure and the Organization of Neural Retina

**Figure 1:**
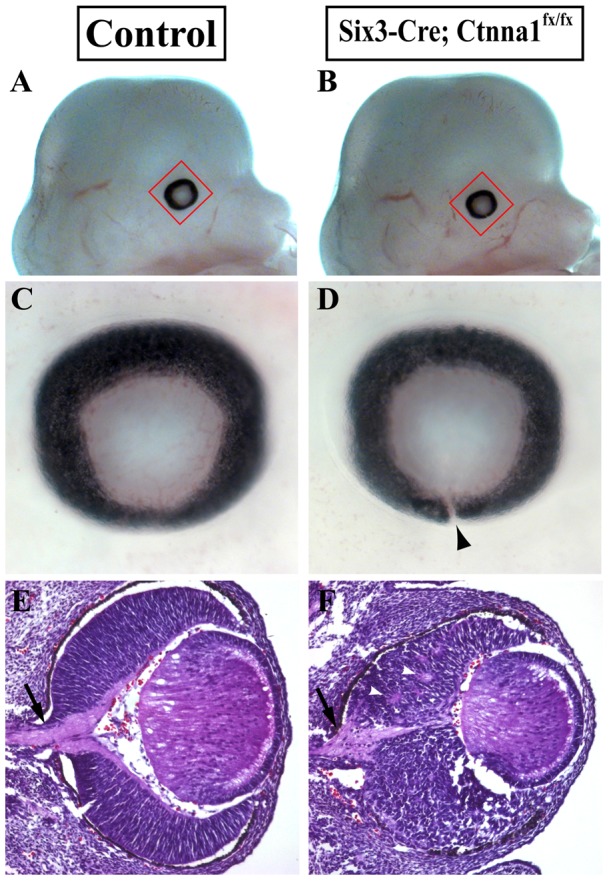
*Six3-Cre; Ctnna1^fx/fx^* mice develop coloboma. (**A**, **B**) E13.5 control and *Six3-Cre; Ctnna1^fx/fx^* mouse heads. (**C**, **D**) The eyes highlighted in **A** and **B** are shown at higher magnification. The unclosed OF is indicated by an arrowhead in **D**. (**E**, **F**) Hematoxylin-eosin stained frontal sections of E13.5 control and *α-catenin* mutant eyes. Black arrows in **E** and **F** point to the optic nerve, whereas white arrowheads denote the cell clusters.

Because homozygous *α-catenin* mutant embryos die at blastocyst stage [32], we thus used a retina specific *Cre* line, *Six3-Cre* [29], and a *α-catenin* conditional allele, *Ctnna1^fx^* [30], to inactivate *α-catenin* specifically in the developing retina. Six3 is a homeobox-containing transcription factor that is expressed in all mouse retinal progenitor cells and the optic stalk (OS) from the optic vesicle stage [33]. *Six3-Cre* starts its expression in the OC and the OS from E9.5 [29]. Because the OF closure finishes at around E12, we thus harvested the *α-catenin* mutant embryos at E13.5 to examine whether there is any defect in the OF closure. At E13.5, the OF in the control mouse has already closed, which is evidenced by the appearance that the eye ball is completely enwrapped by an intact pigmented epithelium (Fig. 1A and 1C). However, in all the *Six3-Cre; Ctnna1^fx/fx^* embryos, a cleft, which is evidenced by the discontinuity of the RPE epithelium, is consistently observed on the ventral side of the eye (Fig. 1B and 1D, n = 10). These results indicate that *α-catenin* is required for the OF closure.

Because N-cadherin-mediated cell adhesion has been shown to be important for the organization of the neural retina in zebrafish [25], we then used hematoxylin-eosin (HE) staining sections to determine if *α-catenin* is also required for the organization of the neural retina. In the control eye, the NR progenitor cells are orderly arranged as a pseudostratified epithelia layer, which is wrapped by the RPE layer (Fig. 1E). The continuity of the retina is only disrupted at the center by the optic disc, which provides the exit for the axons of retinal ganglion cells (Fig. 1E, black arrow). In contrast, the NR layer in the *Six3-Cre; Ctnna1^fx/fx^* eyes is disorganized, with neural retinal progenitor cells aggregated in small clusters (Fig. 1F, white arrowheads). Interestingly, the optic nerve still forms in the *Six3-Cre; Ctnna1^fx/fx^* mouse eyes, but appears to be hypotrophic (Fig. 1F, black arrow). These results indicate that *α-catenin* is required for the proper organization of the neural retina.

### *α-catenin* Is Efficiently Deleted from the NR of the *Six3-Cre; Ctnna1^fx/fx^* OCs

**Figure 2:**
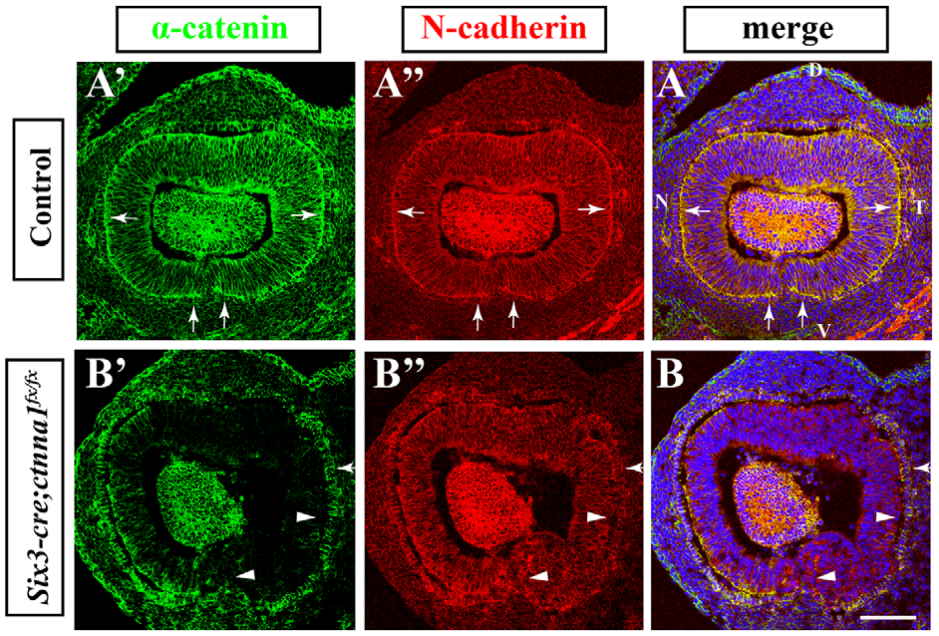
α-catenin is deleted from the developing OCs. (**A**–**A”**) Para-sagittal section of the control OC labeled for α-catenin (**A’**) and N-cadherin (**A”**). Arrows indicate the apical accumulation of α-catenin and N-Cadherin. (**B**–**B”**) Para-sagittal section of the *α-catenin* mutant OC stained for α-catenin (**B’**) and N-cadherin (**B”**). RPE progenitors (arrow) still expression α-catenin, but NR progenitors (arrowheads) lose α-catenin expression and apical N-cadherin accumulation. For all the para-sagittal images of the OCs in this manuscript, the OCs are oriented as indicated in A: D refers to Dorsal, V refers to ventral, N refers to nasal, and T refers to temporal. The scale bar is 100 um.

To determine if *α-catenin* is deleted efficiently in the *Six3-Cre; Ctnna1^fx/fx^* OCs, we used immunohistochemistry to compare the expression of α-catenin protein in E11.5 control and *α-catenin* mutant eyes. One of the major functions of α-catenin in the cell is to connect the AJ to the cortical actin cytoskeleton network, and its absence destabilizes AJs [34]. Thus, we also examined the expression pattern of major AJ components, N-cadherin, together with α-catenin, to determine if AJs are affected in the absence of α-catenin in the developing retina. In the control eyes, α-catenin is abundantly expressed in both the NR and the RPE layer, and it is predominantly localized to the apical side of both the NR and RPE progenitor cells where AJs form (Fig. 2A', arrows). As expected, N-cadherin is also prominently accumulated on the apical sides of both NR and RPE progenitor cells, correlating with α-catenin accumulation (Fig. 2A, arrows). This result reflects the close structural and functional relationship between the two molecules [35]. In the *Six3-Cre; Ctnna1^fx/fx^* eyes, RPE progenitor cells have normal α-catenin expression levels and membrane localization, which is consistent with the fact that *Six3-Cre* is not expressed in RPE progenitor cells (Fig. 2B', arrow). However, NR progenitor cells in the ventral, temporal and dorsal region of the *Six3-Cre; Ctnna1^fx/fx^* eyes have lost α-catenin expression, and those in the nasal region show mosaic deletion patterns (Fig. 2B'). Because the *Six3-Cre* transgene was randomly inserted into the genome [29], its expression is subjected to insertion site variegation, which happens to many transgenes [36]. The incomplete deletion of *α-catenin* in *Six3-Cre; Ctnna1^fx/fx^* eyes is a likely result of mosaic expression of *Six3-Cre*. Although N-cadherin remains expressed in the NR progenitors of the *Six3-Cre; Ctnna1^fx/fx^* OCs, its expression levels in the areas where α-catenin is deleted are lower than that in the control, and more importantly, apical accumulation in the retinal progenitors disappears (Fig. 2B'', arrowheads). These results demonstrate that α-catenin is efficiently deleted from most parts of the NR in *Six3-Cre; Ctnna1^fx/fx^* OCs, including the ventral retina where the OF occurs, and suggest that AJs are disrupted in α-catenin mutant retinal progenitor cells.

### *α-catenin* Is Required for Maintaining Apical AJs and Cell Shape in the NR

**Figure 3:**
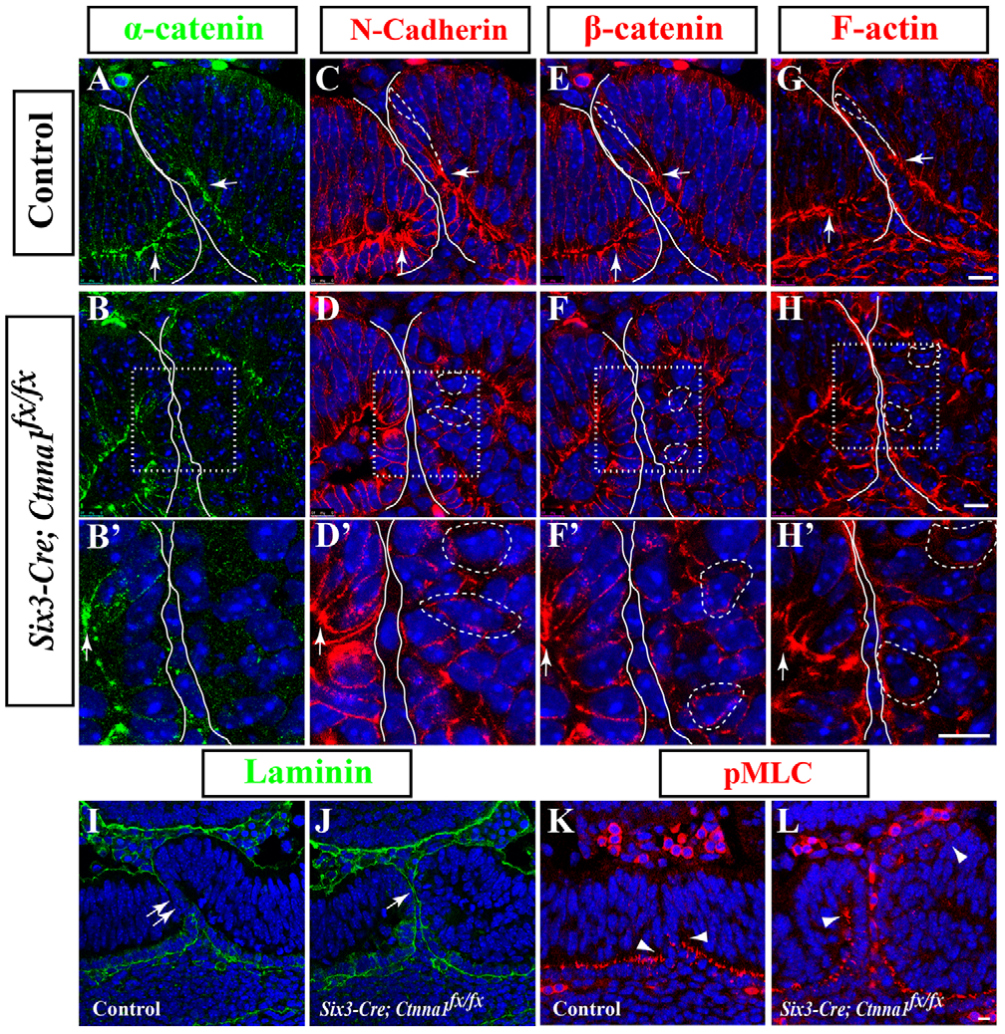
*α-catenin* mutant OFM progenitors show defects in apical localization of AJ components. (A) α-catenin protein expression in the control OFM. (B) α-catenin expression in *α-catenin* conditional mutant OFM. Note that most retinal progenitor cells at temporal OFM (to the right of the image) have lost α-catenin, while many retinal progenitor cells at nasal OFM retain α-catenin expression. (C) N-cadherin expression in the control OFM. (D) N-cadherin expression in the *α-catenin* conditional mutant OFM. (E) β-catenin expression in the control OFM. (F) β-catenin expression in the *α-catenin* conditional mutant OFM. (G) Phalloidin-stained control OFM to illustrate F-actin expression. (H) Phalloidin-stained *α-catenin* conditional mutant OFM to show F-actin expression. (B’, D’, F’ and H’) Higher magnification of squared regions in B, D, F and H. The edges of the OC at the OFM are highlighted by lines to illustrate the OF. Arrows indicate the apical accumulation of AJ components. The morphology of representative retinal progenitor cells at the OFM are highlighted by dashed lines. (I) Laminin expression in the control OFM. Double arrows indicate the fusing OF. (J) Laminin expression in the *α-catenin* conditional mutant OFM. Arrows point to the area where the two sides of the OFMs have moved close to each other. (K) pMLC expression in the control OFM. (L) pMLC expression in the *α-catenin* conditional mutant OFM. Arrowheads in I and J indicate the boundary of pMLC expression. Scale bars are 10 um.

One of the major functions of α-catenin is to connect AJs with the cortical F-actin network, and dynamically regulate the structure and function of AJs [35]. In order to investigate how *α-catenin* mutation contributes to the failure of the OF closure, we first carefully examined the expression patterns of major AJ components in retinal progenitor cells at the OF margin (referred to as OFM hereafter). As we showed earlier, the deletion of *α-catenin* is mosaic in the *Six3-Cre; Ctnna1^fx/fx^* OCs, especially in the nasal retina (Fig. 2). Similarly, most OFM progenitors on the temporal side show an efficient deletion of α-catenin expression, however, most OFM progenitor cells on the nasal retina still retain α-catenin expression (Fig. 3B). Consequently, we have compared AJs in the OFM progenitors between temporal and nasal sides. The retina is a highly organized double-layered epithelium: the progenitor cells in the outer RPE layer are cuboidal-shaped and maintain as a simple epithelium structure, whereas the progenitor cells in the inner NR layer are spindle-shaped, stretching the long axis of their cell bodies across the entire thickness of the layer by keeping their apical and basal ends attached to the basal membrane and the RPE, respectively (Fig. 3C–3G, representative NR progenitor cells are outlined by dashed lines). AJs are specialized intercellular adhesion structures composed of cadherin and β-catenin proteins, and they are connected to the F-actin network through α-catenin [23]. In the control OCs, N-cadherin (Fig. 3C), β-catenin (Fig. 3E), α-catenin (Fig. 3A), as well as F-actin (Fig. 3G) accumulate and form AJs on the apical side of both NR and RPE progenitor cells (arrows in Fig. 3A, 3C, 3E and 3G). In contrast, in the *Six3-Cre; Ctnna1^fx/fx^* OCs, most of the OFM progenitors on the inner layer of the temporal side show a much rounder morphology than their control counterparts, and fails to contact the basal membrane, the RPE, or neither (Fig. 3D, 3F, 3H, and magnified in 3D', 3F' and 3H', representative cells are outlined by dashed lines). Furthermore, AJ components, N-Cadherin, β-catenin and F-actin, are no longer enriched on the apical side of most OFM progenitors on the temporal side (Fig. 3D, 3F, 3H, and magnified in 3D', 3F' and 3H'), but the AJ components are still largely maintained at the apical ends of OFM progenitors on the nasal side due to poor *α-catenin* deletion (arrows in Fig. 3B', 3D' 3F' and 3H'). Thus, our findings on the localization of N-cadherin, β-catenin and F-actin suggest that *α-catenin* mutant OFM progenitors lose AJs on their apical side, and exhibit abnormal morphology.

We next monitored the fusion process of the OF in the *α-catenin* mutant OCs via examination of the integrity of the basal membrane. In the control OC, the OF fusion starts at the folding point where NR and RPE layers meet [10], which is evidenced by the disappearance of the basal membrane (Fig. 3I, double arrows). In the *Six3-Cre; Ctnna1^fx/fx^* OCs, the fusion is never initiated, evidenced by the presence of the intact basal membrane, although two OF margins move close to each other (Fig. 3J, arrow). Because the acto-myosin activity is critical for cell morphological changes and tissue morphogenesis, we next examined the expression of the phosphorylated myosin light chain (pMLC) in the OFM. pMLC represents the active form of the myosin-containing complexes, and the activation of the myosin complexes produce contractile forces or cortical surface tension [37]. Normally, pMLC accumulates at the apical side of the RPE layer, stopping at the folding point at the bottom of the OFM (Fig. 3K, arrowheads). Although the RPE layer maintains normal pMLC expression pattern in the *Six3-Cre; Ctnna1^fx/fx^* OCs, pMLC accumulation extends from the bottom of the OFM to the top of the OFM (Fig. 3L, arrowheads). This abnormal pMLC expression pattern could be the result of the inward projection of the OFM margin or the cause of defective cell morphologies of OFM progenitors.

### α-catenin Is Dispensable for the Specification and Maintenance of OFM and NR Progenitor Fates

**Figure 4:**
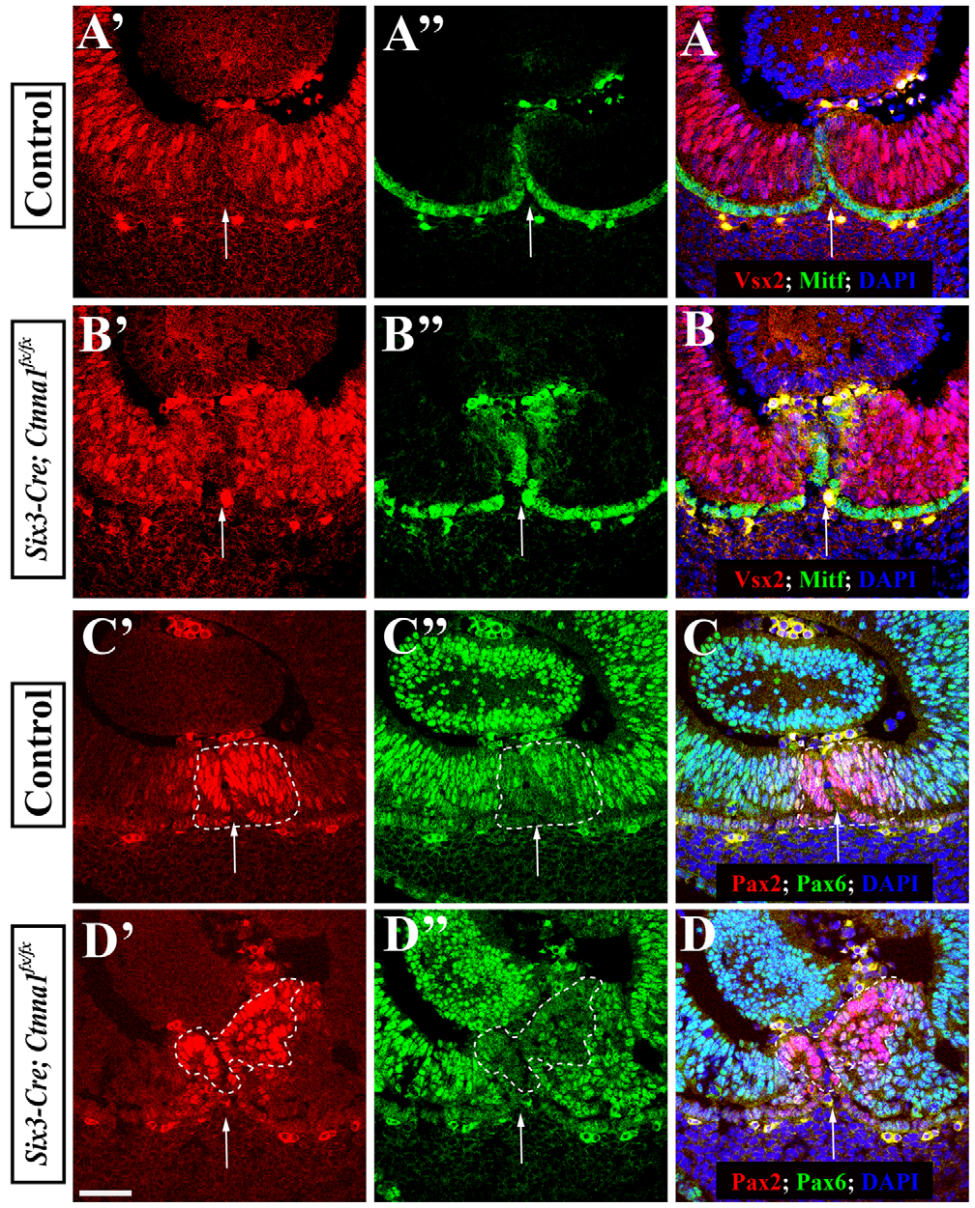
Cell fates are properly specified in the *α-catenin* conditional mutant OCs. (**A’**–**B**) Vsx2 and Mitf expression patterns in the control (**A**) and *α-catenin* conditional mutant (**B**) OFM. (**C’**–**D**) Pax2 and Pax6 expression patterns in the control (C) and *α-catenin* conditional mutant (D) OFM. Dashed lines highlight Pax2-positive OFM progenitors. Arrows indicate the OF. The scale bar is 50 um.

In the developing OC, Vsx2 (previously known as Chx10) and Mitf are specifically expressed in the NR and RPE layers to control their cellular fate and development, respectively. At the control OFM, Vsx2 and Mitf are expressed in the inner and outer layers, respectively, though maybe slightly weaker than in the rest of the OC (Fig. 4A). Even though the *a-catenin* mutant retina shows the obvious organization defect, the progenitors in different regions of the *Six3-Cre; Ctnna1^fx/fx^* retina, including the OFM region, express comparable levels of Vsx2 and Mtif to those in the control retina (Fig. 4B). These results indicate that *α-catenin* is not required for the specification and maintenance of NR and RPE progenitor cell fates.

Pax2 is essential for the OF closure in both humans and mice [12], [38], [39]. Pax2 is highly expressed in OFM progenitors in the control OCs (Fig. 4C'), while Pax6 is only weakly expressed in the OFM progenitors in comparison with NR progenitors (Fig. 4C''). The distinct Pax2 and Pax6 expression patterns result from their reciprocally repressive relationship [40]. Pax2 and Pax6 expression patterns in the *Six3-Cre; Ctnna1^fx/fx^* OCs are similar to those in the control OCs (Fig. 4D). However, we do observe that strong Pax2-positive mutant OFM progenitors are displaced inward toward the lens in the *Six3-Cre; Ctnna1^fx/fx^* OCs (Fig. 4D). Taken together, these results show that α-catenin is not required for the specification and maintenance of the OFM progenitor fate.

### α-catenin Is Dispensable for the Proliferation of OFM Progenitors

**Figure 5:**
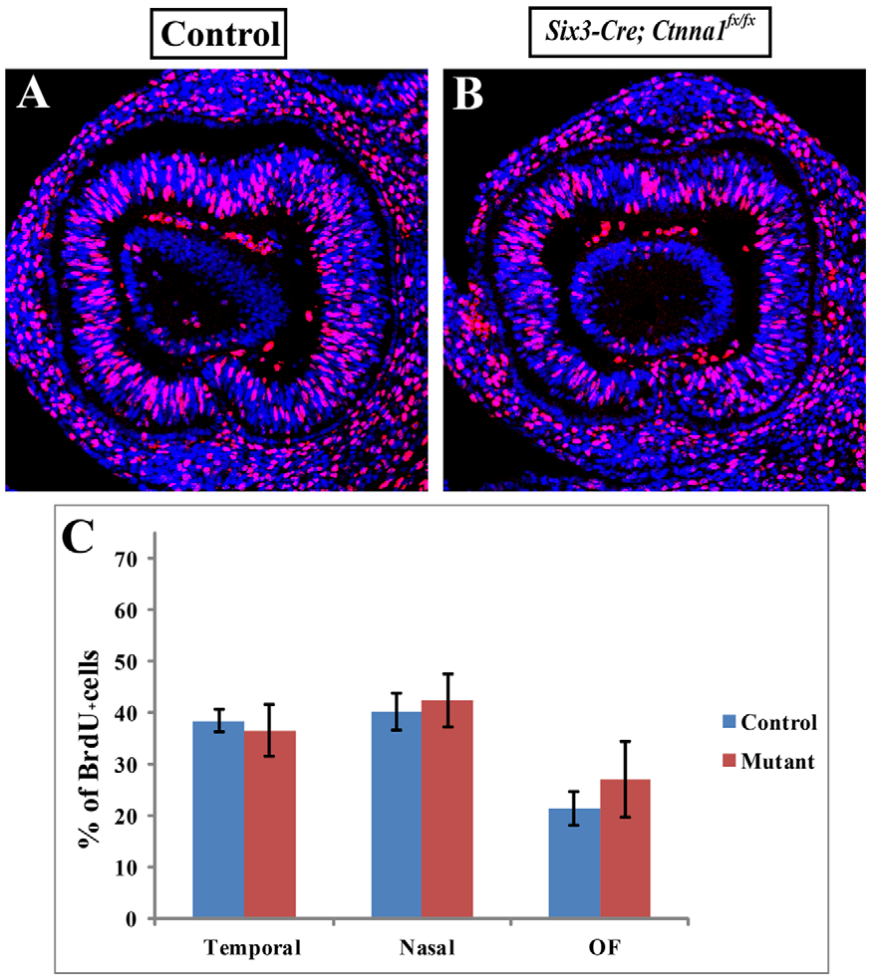
*α-catenin* mutant retinal progenitors proliferate normally. (**A**, **B**) BrdU-labeled sections for control (**A**) and *α-catenin* conditional mutant (**B**) OCs. (**C**) Quantification of BrdU-positive progenitors in the control and conditional mutant retinas.

α-catenin not only regulates cell-cell adhesion, but also has been shown to regulate cell proliferation through modulating signaling pathways [30], [41]. We thus used the BrdU incorporation assay to test whether *α-catenin* also regulates cell proliferation in the retina. Two hours after BrdU was injected into pregnant female mice intraperitoneally, the embryos were harvested for detection of the BrdU label by fluorescent immunostaining. At E11.5, NR progenitors in nasal and temporal regions proliferate actively at similar rates, but OFM progenitors proliferate relatively slower than those in the other regions (Fig. 5A and 5C). In the *Six3-Cre; Ctnna1^fx/fx^* retina, the progenitors in different regions show similar BrdU incorporation rates to their counterparts of the control (Fig. 5B and 5C). These results indicate that α-catenin is dispensable for the proliferation of the progenitors in the developing retina.

### The Retina in the *Six3-Cre; Ctnna1^fx/fx^* mice Degenerates

**Figure 6:**
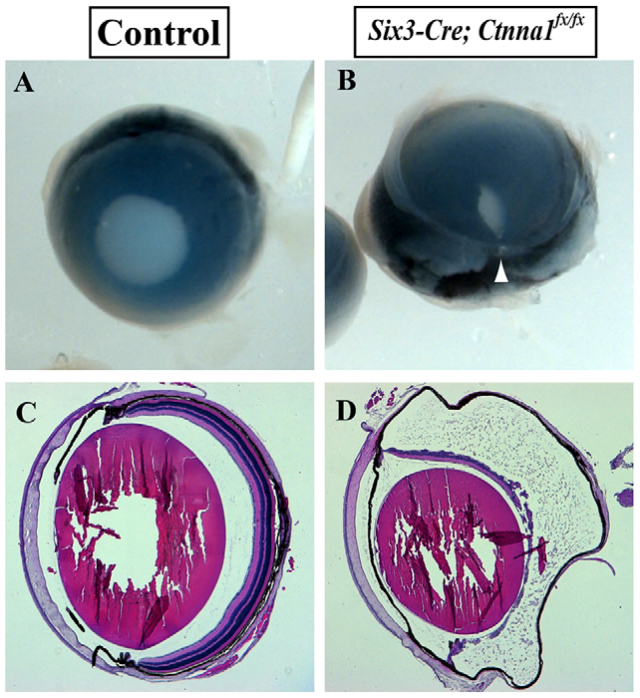
The unclosed fissure persists and the retina degenerates in the *Six3-Cre; Ctnna1^fx/fx^* mice. (**A**, **B**) Eyeballs of 4-month old control (**A**) and *α-catenin* conditional mutant (**B**) mice. The unclosed OF is indicated by an arrowhead in B. (**C**, **D**) Hematoxylin-eosin stained cross-sections of 4-month old control and *α-catenin* conditional mutant eyes. Note that the mutant retina (**D**) is much thinner than the control retina (**C**).

The control adult eye balls show a perfect round pupil in the center (Fig. 6A). Histologically, the adult retina is finely organized into three distinct cellular layers (Fig. 6C). In contrast, the *Six3-Cre; Ctnna1^fx/fx^* eyeballs have a smaller pupil, which is shifted to the ventral side of the eye, suggesting that the mis-organized mutant retina may affect the development of a normal eye structure or the development of the iris (Fig. 6B). In the mutant eyeballs, the open fissure still persists and is visible on the ventral side (Fig. 6B, white arrowhead). Surprisingly, the mutant retina degenerates in the adult mice (Fig. 6D). These results indicate that the unclosed fissure is persistent into adulthood and the retina degenerates in the adult *α-catenin* mutant eye.

## Discussion

Although coloboma is a congenital birth defect that contributes significantly to childhood blindness, the cellular mechanisms underlying the defect are largely unclear. Although cadherin-mediated cell adhesion has been shown to be important for driving the OF closure in the fish, it remains unclear if it is also involved in the regulation of the OF closure in mammals. In this study, we show that a conditional deletion of *α-catenin* from the developing OC results in coloboma formation in mice. Furthermore, we show that *α-catenin* mutant retinal and OFM progenitors fail to form AJs on the apical side, and exhibit abnormal cell morphologies and tissue organization, suggesting that α-catenin-mediated cell-cell adhesion is important for the successful closure of the OF.

As an F-actin binding and bundling protein, α-catenin plays important roles in regulating the assembly and dynamics of actin-cytoskeleton network [42] Through its direct binding to β-catenin, it dynamically connects classic cadherins to the cytoskeleton network, and thus is essential for cadherin-mediated cell adhesion [27], [28], [32]. Consistent with its established roles in the regulation of cadherin-mediated cell adhesion and actin cytoskeleton dynamics, *α-catenin* mutant retinal progenitors fail to form AJ foci on their apical side, which are likely responsible for their abnormal cell morphology, detachment from the basal membrane and the retina disorganization. In the process of the OF closing, the progenitor cells at the OF margin need to coordinate their cell shape changes to ensure that the two sides of the OF are aligned perfectly for the proper fissure closure. Such abnormal cell morphology and organization of the *α-catenin* mutant OFM progenitors likely causes the misalignment between two OF margins and thus the OF closing defect. Although α-catenin has been suggested to modulate different signaling pathways and thus cell fate and proliferation [35], it appears to be not required in OFM progenitors for controlling cell fate determination and cell proliferation. We show that all the major cell types, including NR, RPE and OFM, are properly specified in the *Six3-Cre; Ctnna1^fx/fx^* OCs, and that they also proliferate properly. Based on our experimental findings, we propose that α-catenin primarily regulates cadherin-mediated adhesion and possibly actin dynamics in OFM progenitors, which collectively contribute to the OF closing process.
